# Emergency Department Discharge Instructions: Lessons Learned through Developing New Patient Education Materials

**DOI:** 10.1155/2012/306859

**Published:** 2012-05-15

**Authors:** Danielle M. McCarthy, Kirsten G. Engel, Barbara A. Buckley, Victoria E. Forth, Michael J. Schmidt, James G. Adams, David W. Baker

**Affiliations:** ^1^Department of Emergency Medicine, Feinberg School of Medicine, Northwestern University, 211 E. Ontario St., Suite 200, Chicago, IL 60611, USA; ^2^Institute for Healthcare Studies, Northwestern University, Chicago, IL 60611, USA; ^3^Division of General Internal Medicine, Department of Medicine, Northwestern University, Chicago, IL 60611, USA

## Abstract

Our multidisciplinary team developed a new set of discharge instructions for five common emergency department diagnoses using recommended tools for creating literacy-appropriate and patient-centered education materials. We found that the recommended tools for document creation were essential in constructing the new instructions. However, while the tools were necessary, they were not sufficient. This paper describes the insights gained and lessons learned in this document creation process.

## 1. Introduction

Ninety million Americans have difficulty understanding their own medical care [1]. A large number of studies document that health-related materials exceed the average users reading ability [2]. Within the emergency medicine literature, studies have demonstrated that emergency department (ED) populations are particularly at risk for limited literacy and numeracy [3, 4]. Adding to the problem, print discharge instructions are not written at appropriate reading levels [5–7] and ED patients frequently do not understand their discharge instructions [6, 8–11].

The decision to discharge a patient from the ED is complex and multifactorial; however, once that decision has been made, it is important that patients understand how to properly continue their care at home. In comparison to other aspects of their visit, ED patients have more difficulty understanding their discharge instructions and home care plan than any other component of their visit [12]. In fact, several studies have evaluated patient comprehension of discharge instructions in the days following discharge and have found that up to 78% of patients has an incomplete understanding of their instructions [11, 12]. The patient's ability to comprehend instructions has many implications, not only for the health of the individual, but also for the healthcare system, as patients with poor comprehension are at increased risk for adverse events and increased healthcare utilization [13]. Unfortunately, the high rates of poor comprehension have not encouraged significant changes in the printed materials provided by the ED [14].

Nearly every discharged patient receives instructions upon leaving the ED, but these instructions vary widely. The instructions may be hand written or preformulated. The instructions might be based on an individual physician's notion of “what a patient needs to know” or may have been created by a third-party commercial entity. Regardless of the source of information, individual ED clinicians have no simple means of assessing if a patient will understand the content, if the content addresses the patient's key questions, or even if the patient will read the piece of paper.

In 2010, emergency medicine investigators with expertise in healthcare communication received a foundation grant that supported creation of new discharge instructions to address these concerns. The project engaged a multidisciplinary team of physicians, nurses, healthcare communication and health literacy experts, and patients to develop new discharge instructions. This project used an iterative process of expert review and patient feedback. The rationale for this methodology stems from recommendations by the National Institutes of Health (NIH), National Cancer Institute (NCI), and Center for Disease Control (CDC) for developing print materials and is derived from communication, health education, and health literacy research and practices [15–17]. Similar processes have been recommended in the past for development of outpatient education materials to facilitate behavior change [18] and for the revision of existing outpatient information handouts [19].

Over the past year, this multidisciplinary research team developed a new set of discharge instructions using the recommended tools for creating literacy appropriate and patient-centered education materials. In order to focus the initial effort, five sets of discharge instructions were initially targeted for development. The diagnoses were head injury, kidney stone, ankle sprain, low back pain, and laceration care. We found that the recommended tools for document creation were essential in the construction of the new instructions; while these tools were necessary, they were not sufficient. There were key limitations of the tools, providing valuable lessons. The insights gained through the process are shared in this paper so that those who develop materials in the future may benefit from our experience. Outcomes assessments of the implementation of the documents are ongoing and will be reported separately.

## 2. Less Is More

Patient education materials often attempt to be comprehensive, rather than limiting the content to two or three critical concepts as recommended for those with low literacy skills [20]. Patient comprehension is closely tied to the “working memory constraints” of the reader and short, focused messages are retained better than long, complex messages. This cognitive factors approach, supported by cognitive psychology and learning science principles, improves retention of knowledge from print materials regardless of patient literacy level [21]. Reducing the readability (or reading grade level) of a document is an important first step to improve comprehension but is not enough. Therefore, the best route to increasing information transfer and comprehension is to greatly minimize text. By removing extraneous words and potentially distracting messages, the patient is more likely to retain key information [21].

Simply put, a cognitive factors approach to print materials design is “limit the number of messages.” This task is easier said than done. In the context of our project, we set out to limit the number of messages through an iterative process. We first conducted an electronic survey at our institution to ask all emergency medicine (EM) clinicians to list the five points they felt were essential for a patient to know with each diagnosis. We then developed a comprehensive database of possible “content” through evidence-based guidelines from EM and specialty literature as well as professional society recommendations. We then gathered stakeholder input from focus groups of senior EM clinicians to narrow the material to the information that providers feel is absolutely necessary for patients to know [18]. Finally, we solicited input from specialty physicians with whom the patient would be seeking follow-up care (e.g., urologist input for kidney stone instructions).

At the end of this process, the study group was left with more information and divergent opinions than anticipated. Many clinicians included details that were “nice to know” and potentially important, but not truly critical to avoid complications (e.g., how to blow more air into an ankle aircast). ED physicians were especially focused on topics that fell under the category of “reasons to return” to care. This segment of the instructions is likely driven by the physicians desire to protect the patient from any complications, as well as the fear of post-ED complications or missed diagnoses. The “reasons to return” segment of discharge instructions is often used to provide medical-legal protection and commercially available instructions state that a “carefully designed discharge instruction should transfer some of the responsibility to the patient” [22]. This can result in exhaustive lists of catastrophic outcomes. These lists of reason to return compete with the need to avoid distraction from primary messages.

The process of focusing other messages and lists was more difficult that anticipated. For example, in the case of a kidney stone, how important is it to know to return for signs of infection, uncontrollable pain, or decreased urination? Are these “reasons to return” more important than including “feeling faint,” “increased blood in your urine,” or “vomiting”? Historically, many kidney stone return instructions included information aimed at a missed diagnosis of an abdominal aortic aneurysm or dissection (feeling faint, increased abdominal pain, or having color changes or weakness in the legs). In this era of increased use of CT scans, how frequently are aortic aneurysms missed in the presentation of flank pain and does this information need to be included to avoid bad patient outcomes? Anyone who develops discharge materials is faced with making very difficult and weighty decisions about what information to include and what to exclude. Patient needs and scientific guidance regarding human cognition compete with legal and presumed safety needs. Ultimately, to adhere to the goal of limiting messages and making the documents appropriate for a low-literacy audience, we had to exclude some “reasons to return.” Our guiding principle in this effort was to include the information that was related to the most frequent complications of the diagnosis and information that patients may need to be prompted on to return to care. For example, we did not include detailed information about urinary retention because as any ED clinician can tell you, if a patient is truly in urinary retention, they do not need a prompt to come to the ED, they arrive there very quickly on their own. Ultimately a decision was made that is presumably patient centered. Information was limited to maximize the likelihood of patient understanding of that which was communicated.

## 3. Simple Is Hard

In choosing to target a low literacy audience with our new instructions, we adopted a “universal precautions” approach in the creation of the documents [19, 23, 24]. Creating a document with “plain language” involves an evidence-based approach to structuring, writing, and designing a document that is often confused with “dumbing down” the content [15]. Rather than simplifying the document, using a plain language approach is a means of engaging the audience and making the document more accessible. This approach draws from many fields, including reading, adult learning theory, cognitive psychology, health education and health behavior theories, social marketing, and document design [25]. It is also important to avoid using negative language because patients often misremember negatively worded statements [26].

In addition to thoughtfully designing the word choice of the document, the plain language initiative also emphasizes displaying and organizing the material clearly. Patients read instructions to get answers. Therefore, document design should respond to their questions. In the context of ED discharge instructions, a section heading such as “Diagnosis” was converted to “What is wrong?” to better coincide with the patients' information needs.

We quickly learned that plain and simple language is anything but simple. This was among the most time intensive parts of our document creation process. Through collaboration with the Health Literacy and Learning Program and the Division of General Internal Medicine at Northwestern University, we edited and reedited the documents. The difficulty in using simple language is that it is foreign to healthcare professionals. Every word needs to be carefully chosen and evaluated for the potential for misunderstanding. It is not simply about achieving a lower grade-level score using a literacy calculator. Creating a simple language document requires critical evaluation of each sentence for removing extraneous words, creating simple and parallel sentence constructions, and avoiding negative wording or passive voice. For example, the initial description of a laceration as suggested by the clinicians was “a cut through the layers of your skin and underlying tissues.” Is it necessary for the patient to know about the “underlying tissues” or does that lead to distraction (what are “tissues” anyways?). This definition was initially changed to the statement “a laceration is a cut that goes through all of the layers of the skin.” Does the patient need to know that the cut goes “through all layers of the skin”? That is a lot of words and likely wasted space and memory since it does not provide needed or actionable information. The final version of the sentence reads “you have a deep cut in your skin that needed sutures (stitches) to close it.”

Editing and reediting these documents taught us that using simple language is incredibly difficult. It has also caused us to reflect on the verbal information that we provide to patients on a daily basis as clinicians. Knowing how laborious it is to achieve plain language documents, it is difficult to believe that any clinician is able to use accurate, simple language when verbally interacting with patients on a daily basis.

## 4. A (Good) Picture Is Worth 1000 Words

Creating visuals and maintaining adequate white space is essential for patients to be receptive, engaged, and attentive to the written instructions. Avoiding lengthy text is necessary in order for most patients to read the document. The preservation of white space and incorporation of images proved to be valuable. Well-placed visuals emphasize important points and help to explain the text. The specific choice of visual (line drawing versus cartoon versus photo) depends on the concept being illustrated. Regardless of the type of visual, it should be professional and focus on one message [17]. Illustrations and pictographs improve the comprehension and recall of both written and spoken instructions [27–29].

In entering into this document creation process, time and money had been earmarked for an illustrator to create pictographs. We had the resources to add images to the documents, but then were left wondering, “pictures of what?” In keeping with the tenets described above, we did not want pictographs that were distracting or simply attractive but did not impart key information. In the case of back pain, we considered pictures of a person bending, or drawing of a vertebral column, but questioned whether these images aided essential knowledge. Does a cartoon picture of a person with a bleeding cut on their arm add any value to information about lacerations? In contrast, pictographs can be especially useful in introducing or explaining new or difficult to understand concepts.

After much discussion, we decided to have the illustrator focus on figures that would help to explain potentially difficult terms to the patient. In the ankle sprain instructions, the concept of a “ligament” is introduced by saying “a sprain is a stretch or partial tear of ligaments.” The instructions go on to briefly define a ligament as a “thick band that holds bones together”; however, even with this explanation, “ligament” is a medical jargon term that many patients did not intuitively understand. Therefore, we asked the illustrator to create a simple drawing with labels of bone and ligament to visually depict a ligament. Patients provided key input regarding which images were needed and how they were perceived. For example, the image of the ankle ligament was better understood when the full foot, including toes, was present. Ultimately, patients expressed great satisfaction at the improved understanding about what an ankle sprain actually is.

We do not have illustrations for all five of the diagnoses. This was a point of contention because it was felt by some that patients would respond better to sets of instructions that included illustrations. This was a difficult decision. Should we try to increase the appeal of the document by adding an attractive but space-occupying illustration without a clear purpose? Or would the benefits of increased visual appeal be negated by distracting from the main messages? We felt that pictographs with no essential value would only serve as distracters, and we did not include illustrations in all of the instructions. Ultimately, it was a judgment call, and it is difficult to know if there is a correct answer, but lay participants ultimately supported the decision. Entering this process, we had no idea how animated a discussion of cartoons would become.

## 5. Clarity Is in the Eye of the Beholder

At this point in our research process, we had created working drafts of the discharge documents for all five diagnoses. We were proud of these documents and had confidence that our efforts had anticipated many of the problems that patients with low literacy typically experience with written education materials. Known communication standards had been followed, and additional unanticipated challenges had been thoughtfully addressed. However, we wanted to test our documents with real patients and receive feedback before “going live.” Directly involving patients in the creation and revision of health education materials is valuable because it highlights potential areas of mismatched understanding [30]. Patient involvement also ensures that the presentation of information is authentic, relevant, and presents the information from the perspective of the learners [31].

To obtain this perspective, we conducted focus groups for each of the discharge documents. Patients recruited from local federally qualified health centers participated in the five focus group sessions. Audiorecordings of the groups were analyzed using latent content and constant comparative analysis to code for emergent themes [32]. Several important themes were uncovered (full results reported separately), and the documents were adjusted in response to patient feedback [33]. For example, focus group patients wanted to know more details about specific recommendations for home care. In the setting of ankle sprain, the recommendation to “apply ice” was expanded upon read “apply ice for 10–15 minutes, repeat in 1-2 hours and continue as often as possible for 2 days.” The participants said “tell me what I need to do” and “tell me when I am going to feel better.” It was less clear to patients why there were instructions about “return to the emergency department” since they believed that the evaluation had been done and the diagnosis was known. Interestingly, patients also requested that we define why they should follow instructions, asking “what is in it for me?” which led to the inclusion of the following statement: “the following are things you can do to feel better faster.” Finally, the patients also made recommendations on which concepts they felt needed to be accompanied by an illustration.

This process was humbling. Our document was dramatically modified. Despite our best efforts to anticipate the patients' perspective, including having nonmedically trained research assistants and coordinators at all document creation meetings, there was still significant room for improvement. We would strongly encourage that any patient education materials incorporate a patient feedback step into their document creation process because it provided us with invaluable insight.

## 6. Beauty Is Only Skin Deep (but It Still Matters)

After incorporating the focus group recommendations, we wanted to take the documents back to patients again. Before taking that step, we performed several analyses on the documents to ensure their suitability. A Suitability Assessment of Materials (SAMs) evaluation grades print materials as “not suitable,” “adequate,” or “superior” after completion of an evaluation in six categories (content, literacy demand, graphics, layout, learning stimulation, and cultural appropriateness) [20, 34]. Using standard methods for the SAM assessment, three independent trained raters performed a SAM assessment of both the newly created documents and of the commercially available instructions used at our institution. The results of the SAM assessments are shown in Table 1. For all five diagnoses, the newly created documents were rated more suitable than the commercially available documents.

With the evidence in hand that our documents were more “suitable,” we then moved on to performing individual cognitive interviews with patients in the ED. Sixty patients were enrolled (mean age 48; 73% female; 67% Caucasian, mean REALM score 63.6 (9th grade and above)) to evaluate our new instructions for back pain and ankle sprain. Patients were given a copy of our new instructions, as well as a copy of the commercially available instructions for the same diagnosis. Overall, patients preferred our new instructions and ranked them higher in overall understanding, preparation for home care and clarity. Interestingly, despite our newly created documents receiving higher ratings for both preference and clarity, when patients were asked which document they would prefer to take home, they selected the commercially available products. The patients said that the commercially available documents were more visually appealing. The newly created documents had not been specially formatted; they were simply printed out as a document. Potentially more important, the patients said that they also selected the commercially available documents because there seemed to be more information.

This result was disappointing. We learned that beauty is only skin deep, but it still matters. Despite all of our efforts and despite the patients acknowledging that our document was superior in several respects, they still did not prefer it. We felt that all of the hard work had been accomplished in terms of the content of the instructions and it was a disappointment that our work was not rewarded because of the lack of layout formatting and graphic design. However, there was an easy fix—make the documents pretty. In light of the feedback received from this round of testing, the study group commissioned a series of graphic designers to enhance the documents. The study group reviewed approximately twenty different designs and selected the five design formats that were most consistent with the tenets of creating clear documents outlined above. A group of fifty patients and providers then ranked the design formats from favorite to least favorite, and a final design template was chosen.

## 7. Conclusions

**Figure 1:**
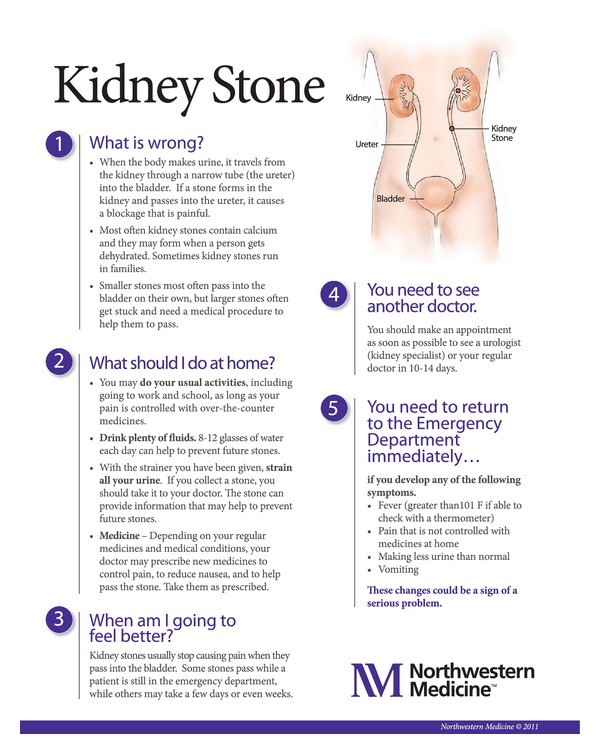
Revised kidney stone discharge instructions.

A set of discharge instructions was created for five diagnoses that conform to existing healthcare literacy guidelines and overcome newly identified hurdles (Figure 1). In final testing, the newly created instructions were preferred by patients and caregivers alike. By focusing materials on “need to know” information and limiting the documents to essential content, the documents teach to the learning needs of the audience and avoid information overload. Preliminary evaluation of the documents through patient focus groups, comprehension testing, and SAM assessments indicates that the documents created through this process are superior to the discharge documents currently utilized at our institution. The documents impact on patient comprehension and behavior following an ED visit will need to be further assessed. Completion of patient comprehension assessment and formal assessment of impact on medical outcomes such as revisit rates, complications, and subsequent resource utilization is planned. In the future, these documents will serve as the foundation for a comprehensive discharge process, including written, verbal, and follow-up components, and our research team plans to rigorously evaluate their impact on downstream outcomes including comprehension, adherence, and resource utilization.

Although the final *outcomes* of our research are not currently available, we learned many unexpected lessons through the document creation *process *that may be beneficial to others. We entered the process armed with toolkits and guidelines that were invaluable; however, those resources only took us part way. We were faced with unanticipated difficult decisions in narrowing the content. Simple was anything but simple in crafting the language. We started the process focused on improving the written content but later learned that equal weight needs to be given to illustrations and “look.” We were humbled repeatedly as we learned from our patients how to consider information from their perspective. Finally, this was a learning experience for the clinician researchers who learned key lessons regarding patient-oriented communication, specifically in simplifying language, focusing messages, using natural speech, and understanding the way that patients perceive or misperceive the intention of the communication. Hopefully, future researchers targeting patient education materials can also benefit from our experience and enter their own projects better armed to tackle these challenges.

## Figures and Tables

**Table 1:** Suitability Assessment of Materials (SAM)^#^.

Diagnosis	Commercially available		New	
	Score/possible points	Suitability rating*	Score/possible points	Suitability rating*
Laceration care	9/30 (30.0%)	Not suitable material	22/30 (73.3%)	Superior material
Kidney stone	8/38 (21.0%)	Not suitable material	21/38 (55.3%)	Adequate material
Head injury	9/38 (23.7%)	Not suitable material	18/30 (60.0%)	Adequate material
Back pain	7/38 (18.4%)	Not suitable material	19/30 (63.3%)	Adequate material
Ankle sprain	13/38 (34.2%)	Not suitable material	24/38 (63.2%)	Adequate material

^#^Number of possible points varies (30 for items without illustration, 38 for items with illustrations); interrater agreement 81.61%.

*The percent score correlates to three suitability ratings: 0–39%: “not suitable material”; 40–69%: “adequate material”; 70–100%: “superior material.”
